# The effects of vocational interest on study results: Student person – environment fit and program interest diversity

**DOI:** 10.1371/journal.pone.0214618

**Published:** 2019-04-04

**Authors:** Stijn Schelfhout, Bart Wille, Lot Fonteyne, Elisabeth Roels, Filip De Fruyt, Wouter Duyck

**Affiliations:** 1 Department of Experimental Psychology, Faculty of Psychology and Educational Sciences, Ghent University, Ghent, Belgium; 2 Department of Personnel Management, Work and Organizational Psychology, Faculty of Psychology and Educational Sciences, Ghent University, Ghent, Belgium; 3 Student Counseling Office, Department of Educational Policy, Campus UFO, Ghent University, Ghent, Belgium; 4 Department of Developmental, Personality and Social Psychology, Faculty of Psychology and Educational Sciences, Ghent University, Ghent, Belgium; Kyoto University, JAPAN

## Abstract

The extent to which a good person-environment (PE) interest fit between student and study program leads to better study results in higher education is an ongoing debate wherein the role of the study program environment has remained inadequately studied. Unanswered questions include: how diverse study programs are in the interests of their student populations, and how this program interest diversity influences study results, in comparison to individual PE fit? The present study addressed these questions in students (N = 4,635) enrolled in open-access university education. In such an open access system, students are allowed to make study choices without prior limitations based on previous achievement or high stakes testing. Starting from the homogeneity assumption applied to this open access setting, we propose several hypotheses regarding program interest diversity, motivation, student-program interest fit, and study results. Furthermore, we applied a method of measuring interest diversity based on an existing measure of correlational person-environment fit. Results indicated that interest diversity in an open access study environment was low across study programs. Results also showed the variance present in program interest diversity was linked to autonomous and controlled motivation in the programs’ student populations. Finally, program interest diversity better explained study results than individual student fit with their program of choice. Indeed, program interest diversity explained up to 44% of the variance in the average program’s study results while individual student-program fit hardly predicted study success at all. Educational policy makers should therefore be aware of the importance of both interest fit and interest diversity during the process of study orientation.

## Introduction

Literature has shown that students who choose a study program that fits their vocational interests, arguably have better study results and have a better chance of finishing higher education in a timely fashion [1]. Such study results are usually investigated in settings without much emphasis on the educational access policy. However, these policies show large variety: they can be open or restricted, based on past secondary school performance or tests like the SAT (scholastic aptitude test). This person-environment fit (PE fit) research line focuses heavily on the student side of the PE relationship, while leaving the environment of study programs underexplored [2]. As a consequence, it is still unknown how diverse study environments actually are in terms of student vocational interest, and whether this varies as a function of access policy. For instance, does an academic bachelor in psychology only attract students that are profoundly interested in psychology or do students who enroll in psychology display quite some diversity in their interest pattern? And how does interest diversity in a psychology program compare to the diversity in other programs like mathematics or economics? As literature is still oblivious of study program interest diversity, we are also unsure how this program diversity directly influences study results. In an open access educational environment, the present study uses homogeneity theory and the properties of vocational interest to pose and answer three main research questions. How diverse are student interests within and between study programs? Does this interest diversity directly influence average study results in study programs? And finally, how does this effect of program interest diversity on study results compare to the effects of individual PE fit?

### The properties of vocational interest

Vocational interest is typically defined as the liking or disliking of certain activities or environments, usually represented by a concise number of dimensions [3]. Vocational interest also has a number of key characteristics which are important when exploring the relation between student interest and study results [4,5].

First, vocational interest has predictive power towards study program choice [6]. Up to 70% of the students (depending on the methodology used) chooses a study program that can be predicted through vocational interest [7,8]. As such, a vocational interest model should be able to compare student interest profile and study program environment on a commensurate scale to explore how good students match their study choice [9]. For the present study, we have used the RIASEC model by Holland [10].

Next, student interests always have an object or an environment [11]. As an example, a student can be interested in solving equations or working in an engineering environment. As a consequence, questionnaires targeting higher education students should focus on appropriate items like activities or (future) occupations. For this specific study, we have used the SIMON-I questionnaire [12], but our rationale could be easily applied to any other RIASEC-based instrument.

Literature also reports that vocational interests are stable constructs [13]. This opens up research possibilities towards prospective studies. As an example, in the present study we have combined students’ interests and study results spanning an entire academic year.

Finally, interests are also linked to motivation [14]. Performing actions which the student is highly interested in, like solving equations, can create a study environment that motivates the student towards obtaining his or her degree of choice through facilitating study behavior. Because motivation and vocational interest are linked together, we expect that average program motivation scores of student populations have also been linked to the interest diversity in student populations. As such, we have also assessed both controlled and autonomous motivation in the present study. Autonomous behavior is performed out of interest or personal importance, while controlled behavior is driven by external demands [15]. For instance, a student can get good grades because he likes studying the courses in his program of choice (autonomous motivation) or because he wants to adhere to his parents’ expectations (controlled motivation).

### RIASEC theory

The Holland RIASEC model has a long standing tradition as one of the most influential models of vocational interest [16,2,17]. The RIASEC theory’s basic principle is very straightforward. Persons (students) and environments (study programs) are represented on the same clockwise hexagon, containing six dimensions or scales, using the same RIASEC code: *Realistic*, *Investigative*, *Artistic*, *Social*, *Enterprising*, and *Conventional*. By comparing the profiles of a student and a study program, PE fit indicates how well a (future) student’s interests match a specific study program. The current iteration of the RIASEC model still instigates a lot of research and applications specifically targeting (higher) education. As an example, the current study builds on the SIMON-I RIASEC questionnaire that was recently introduced as a study orientation tool, tailored for the transition towards higher education [12]. SIMON-I measures the interests of (future) higher education students in order to guide the student towards the best fitting study programs. As such, SIMON-I builds on the object characteristic of vocational interest by including items that describe professions (66) and activities (87), tied to one of the six scales. As an example, *Geneticist*? (I) or *Starting up an enterprise*? (E) are two of a total of 153 very short items to which the (future) student had to answer with yes or no. The scores on all dimensions can be recalibrated to a score on a 0–100 scale for each dimension, effectively rendering a student RIASEC profile, e.g. R: 80, I: 70, A:60, S:50, E:55, C:59.

Beside these person profiles, there are a number of ways to construct RIASEC study program environment profiles. One of these methods is built on a common principle that the environment is determined through the people that are in it [18–20]. As vocational interest is a stable construct, students having a good fit with their study programs in year one will likely still have a good fit when they finish their study program. By using successful and persistent students as representatives or *incumbents* of a study program, this incumbent method can empirically generate environment profiles using the profiles of said students [21,10]. Due to this empirical base, the incumbent method is immune to rater bias, in contrast to profile generation based on expert ratings. Practically, the RIASEC profile of the study program is constructed by averaging out the scores on the RIASEC dimensions using the incumbent student RIASEC profiles of that program [21]. The relation or (dis)similarity between the individual student and the study program environment is depicted through a measure of PE fit.

### Measure of PE Fit: Pearson’s product moment correlation coefficient

Correlational fit is a pattern based similarity measure, that correlates commensurate student and study program RIASEC dimension profile patterns by using the *Pearson’s product-moment correlation coefficient*. As such, correlational fit indicates how well the student’s interests fit with his or her study program of choice. For instance, fitting a study program profile (M) R:80, I:70, A:60, S:50, E:60, C:70 to a student profile (S) R:80, I:60, A:60, S:50, E:50, C:60 results in a correlation of .87 (or vice versa). A high correlation means a better PE fit. Literature has shown that correlational fit also has predictive value towards study results, especially for first year students [21,9].

Although the predictive validity of PE fit between students and their study programs on study results has repeatedly been established, the positive influence of this PE fit on study results remains somewhat limited, varying from a very small to medium effect at best [22–25]. Indeed, some studies report at best a very modest effect of PE fit on study results, while other studies report correlations of up to .32 between study results and PE fit, implying an explained variance of about 10% [26]. As a consequence, a debate is still ongoing whether or not the magnitude of these results are in line with the high(er) expectations of theoretical vocational interest models like the Holland RIASEC hexagon [10].

However, when starting a new study related to concepts like PE fit, researchers are usually confronted with a selection bias problem. Access to study programs, especially in the United States, is often already restricted and linked to performance criteria like passing a specific exam or satisfying grade point average (GPA) requirements. As such, these access restrictions yield a pre-selected sample, possibly not only biased in terms of intellectual competence, but potentially also biased towards PE fit between students and programs. It is therefore a possibility that the variation in reported PE fit effects reflects–at least partly–the variation in educational access policies. The present study provides a unique opportunity to enhance our knowledge on the range of PE fit effects by using a large student data set from an open access higher education environment. It will be very interesting to observe whether the positive effect of PE fit on study results generalizes to, or is even stronger under conditions of free study choice, while also examining the influence of program interest diversity on average program results.

### Interest diversity theory

The age old homogeneity assumption states that people who display similarity in characteristics such as vocational interests tend to lean towards similar environments, with literature spanning more than half a century [27,28,20]. As a consequence, environments like professional occupations are inhabited with individuals that have similar patterns of vocational interests. This internal similarity in the population of an environment also seems to hold in higher education when observing the vocational interest of students. As an example, students that show a good fit between their RIASEC profile and a specific study program are more likely to enroll for such a program than students who do not fit the program [11,6]. Starting from this internal similarity in the population of study programs, we can make a number of predictions. These predictions form the basis for the present study’s research questions.

To start, study programs should display a low interest diversity in their student population. Indeed, individual students who have a high PE fit with a study program are more like to enroll for this specific study program than students who lack a high PE fit. This mechanism will be even more explicit if there are no further requirements (like GPA or exams) to enter a program as is the case in our present study with open access. As a consequence, the average PE fit in our open access study program population should be quite high when compared to programs with a more restricted access. Due to this high individual PE fit across students, each study program should display high internal similarity, or a low diversity, regarding the vocational interest of its student population. In order to be able and test our predictions of open access versus restricted access, the present study also features data from a small control group (same university) that had to pass an entry exam to enter the Medicine or Dentistry programs.

Next, a high interest in a specific program is just one, autonomous motivation why students enroll for that program. Students can also opt to act on exterior, more controlled motives to make their choice, like pleasing their parents. As these students are less interested in the specific program, they will have a lower PE fit with the chosen program. Some study programs might be more prone to such externally controlled study choice than others; these programs will attract more students with a lower PE fit and thus show a higher interest diversity in their student population. For the present study, we therefore predict that program interest diversity will vary over programs. We also predict this variance will be linked to autonomous and controlled motivation.

Finally, the internal similarity of the environment will exert an influence on the behavior of the population, with different programs rewarding different interest patterns [29]. In case of high internal similarity, the behavior of the students will be less determined by their interest pattern but all the more by the study environment. For instance, Tracey and colleagues used the profile deviation around the mean of the six RIASEC dimensions of a study program as a measure of so called constraint [9]. Smaller deviations represent higher constraint and higher internal similarity. Relevant for this study, results showed a cross-level interaction between program constraint and student interest pattern. Indeed, high program constraint reduced the effect of individual PE fit on study outcomes like GPA and persistence. However, their study did not specifically focus on the direct influence of the environment on study results at the program level, nor on the comparison between the effects of the environment (internal similarity) and the effects of the individual (student PE fit). Our study aims to add to this knowledge. Considering the already hypothesized low levels of program diversity (high internal similarity), we predict a strong effect of the environment for the present study.

### Measuring interest diversity

The debate regarding the best fitting (dichotomous) measure for studying homogeneity or internal similarity of environments is still undecided. However, Bradley-Geist and Landis provide evidence that first and foremost, the measures used should depend on the study’s major hypotheses [30]. For the present study, the emphasis lies on study program interest diversity and its influence on study results in higher education. As such, interest diversity should be assessable within study programs. Moreover, a program interest diversity assessment should also allow for comparisons across programs so we can investigate the possible influence on study results. For instance, the average deviation measure for testing environment homogeneity reflects the average difference within one group between the individual scores and the mean or median group score [31,32]. Regarding vocational interest, we are faced with a conceptual problem of measuring such an average deviation on a single scale. Indeed, the most dominant theories use multiple scales to measure vocational interest. For example, our Holland RIASEC theory uses a 6 dimensional hexagon to depict the vocational interest of individuals and their vocational environments like study programs (see above). As it stands, correlational fit already provides us with a one-scale, parsimonious and continuous PE fit measure that indicates the degree of fit of an individual’s interests with his or her environment. Furthermore, a PE fit correlation is already known to be predictive of first year study results [9]. Apart from indicating a degree of fit, the correlational fit measure also indicates how far a student’s interests deviate from their study program interest profile. By averaging out these deviance measurements over students of a specific program, a continuous measure of program environment interest diversity can be obtained. Such a measure allows us to investigate whether study program populations have a high internal similarity in vocational interest, while still allowing for variance in this hypothesized low program interest diversity.

### Present study

In the present prospective study, we have derived and investigated a number of research questions from the theoretical predictions made in the introduction regarding program interest diversity in an open access environment. For our first question, we have investigated how diverse study programs actually are in the vocational interest of their student population. Students with similar interest patterns should be attracted to similar programs, especially in an open access environment. Consequentially, the fit between students and their program of choice should be high. As such, we hypothesize program interest diversity will be low. We also expect that this general low interest diversity will still show variance over the range of programs, linked to the motivation of their student populations. Indeed, some students will choose the program autonomously, because they are interested in the program itself, while others will take into account exterior motives like parental approval and are less interested in the program itself. We expect some programs could be more prone to such exterior motives of student choice. Therefore, the student population of these programs should show more diverse interest patterns. In sum, we hypothesize that a student population with high autonomous motivation is related to a low program interest diversity, while a population with high controlled motivation is linked to a higher program interest diversity.

For our second question, we have explored if and how program interest diversity has an effect on (average) program study results. On an individual level, literature already showed us that a higher PE fit will lead to better study results [9]. One could make the seemingly plausible hypothesis that the program level interest diversity variable derived from this PE fit would show a similar effect, i.e. a lower program interest diversity (high average PE fit of the students) would lead to better results. However, we are wary of making that hypothesis, as we are aware of the ecological fallacy phenomenon that warns researchers to not naively assume that individual effects automatically generalize towards a higher (program) level [33]. Moreover, Smart and colleagues already pointed out that different study programs could reward different interest patterns [29]. As such, we have taken a conservative approach by pitting three hypotheses against each other. We hypothesize rising program interest diversity could show a linear positive effect, a linear negative effect or a curvilinear mixed effect on average program study results. Our findings will also serve as a baseline to integrate possible program interest diversity effects into our third and final research question.

For our final research question, we have compared the found effects of program interest diversity on study results to the effect of individual PE fit. As we are still unsure about the nature of the program interest diversity effect, we can only make predictions regarding individual PE fit. From theory discussed in the introduction, we hypothesize that the individual effects of PE fit on study results will be small, as program environments with low interest diversity (high similarity) are expected to limit the effect of individual PE fit.

## Materials and methods

### Data and procedure

All students (*N*_0_ = 6,772, 55% female) starting an academic bachelor at a large Western European university (ranked in the Shanghai top 100, see also www.shanghairanking.com) across eleven faculties and 41 study programs, with an open access policy (anyone who completed secondary education) were invited to participate in a long term assessment to enhance study choice and study results. Though the programs have an open access structure, they are also strictly stratified. In other words, within one program, everyone has to take the same set of study courses during the first year. The study programs are listed in Table 1. Students were asked to participate in the present study during the starting week (end of September 2016) of their curriculum via their lectors, email and the online learning platform [12]. Response rate was 71% (*N* = 4,827, 57% female). Participating students immediately filled out online interest and motivation questionnaires (about five to ten minutes long, see measures for a detailed description). All students were also subject to periodic evaluation systems (once for each course) split up into two sessions (January and May/June 2017) and a retry session (August/September 2017) if they failed the test on the first attempt. At the end of the academic year (September 2017), the results from the SIMON-I test were cross-referenced to the exam results. A total of 4,422 students (92% of N) at least participated in some form of evaluation. The remaining 8% dropped out, prior to any form of evaluation. As literature shows that PE fit also has an influence on perseverance, we took a conservative approach and included the dropouts in our study [21]. Students from the study programs Medicine and Dentistry (*n* = 192, response rate 86%) already had to pass an entry exam to be allowed to start, in contrast to the students who picked any of the other 39 study programs. As such a high stakes access mechanism (possibly) influences the homogeneity inside a such a program, we have decided to again act conservatively and exclude both programs from our study. However, we have pooled the students from both programs (program RIASEC profiles correlate 0.99 and have about 50% common courses) as a control group for our first question regarding program interest diversity. The final pool of student participants (SP) was *N* = 4,635, spread out across 39 study programs.

**Table 1:** Study programs included in the present study.

Number	Programs
1	Psychology
2	Communication Sciences
3	Mathematics
4	Educational Sciences
5	Political Sciences
6	Law
7	Sociology
8	Criminological Sciences
9	Speech Language and Hearing Sciences
10	Physical Education and Movement Sciences
11	Philosophy
12	Linguistics and Literature
13	East European Languages and Cultures
14	History
15	Oriental Languages and Cultures
16	Moral Sciences
17	Art History
18	Archaeology
19	African Studies
20	Veterinary Medicine
21	Physical Therapy and Motor Rehabilitation
22	Pharmaceutical Sciences
23	Bioscience Engineering
24	Economics
25	Biomedical Sciences
26	Engineering–Architecture
27	Engineering
28	Business Economics
29	Bioscience Engineering Technology
30	Engineering Technology
31	Applied Language Studies
32	Biochemistry and Biotechnology
33	Biology
34	Chemistry
35	Physics and Astronomy
36	Geology
37	Geography and Geomatics
38	Computer Sciences
39	Public Administration and Management
	Medicine and Dentistry (excluded)

Besides this first year student pool, we established the profiles of the study programs using interest questionnaire responses of 6,572 senior 3^rd^ and 4^th^ year students spread out over the same 39 study programs featuring in Table 1. These students all met the conditions of academic success and perseverance. The procedure of making the program E-profiles was identical to the procedure used by Allen and Robbins: the RIASEC scores of all students in each study program were averaged out across all six dimensions to obtain the E-profile for each study program [21].

### Ethics statement

*The Ethical Commission of the Faculty of Psychology and Educational Sciences at Ghent University* has granted approval to "*Constructing Simon*: *a tool for evaluating personal capacity to choose a post-secondary major that maximally suits the potential*." of which the present study is an integral part, with reference 2016/82/Elisabeth Roels.

This study was carried out in accordance with the recommendations of the Ethical Commission of the Faculty of Psychology and Educational Sciences at Ghent University. All subjects gave online informed consent in accordance with the Declaration of Helsinki. The protocol was approved by the Ethical Commission of the Faculty of Psychology and Educational Sciences at Ghent University.

Considering the nature and size of the online project and the highly restricted access to the information of the participants, an explicit written informed consent was deemed unworkable and unnecessary and was replaced with an explicit online informed consent of which a translation is provided below.

Prior to filling out the online surveys, students had to explicitly agree to the following statements: (translation)

#### Processing of personal information

The provided personal information can be linked to study results in higher education. This information can be used for scientific purposes and for counseling in higher education. Personal information will be stored in a separate data file. Under no condition will this personal information be communicated to third parties. Ghent University is responsible for the processing of the information. Participants are not obliged to provide this information, they always have access to this information if they so desire and have the right to correct or adjust this information through SIMON@ugent.be.

#### Copyright

This test and the internet files attached to it are protected through copyright. Test results are allowed to be printed for personal use. Copying, adjusting, translating, editing or changing the whole or parts of this test or site, in any way, shape or form, mechanically or other, are strictly forbidden, unless explicit written permission was obtained.

#### Liability

Though the content of this test was subject of extreme scrutiny, no liability can be accepted for possible errors. (for the student) I agree to these terms: yes no

### Measures

#### Vocational interest

**Fig 1:**
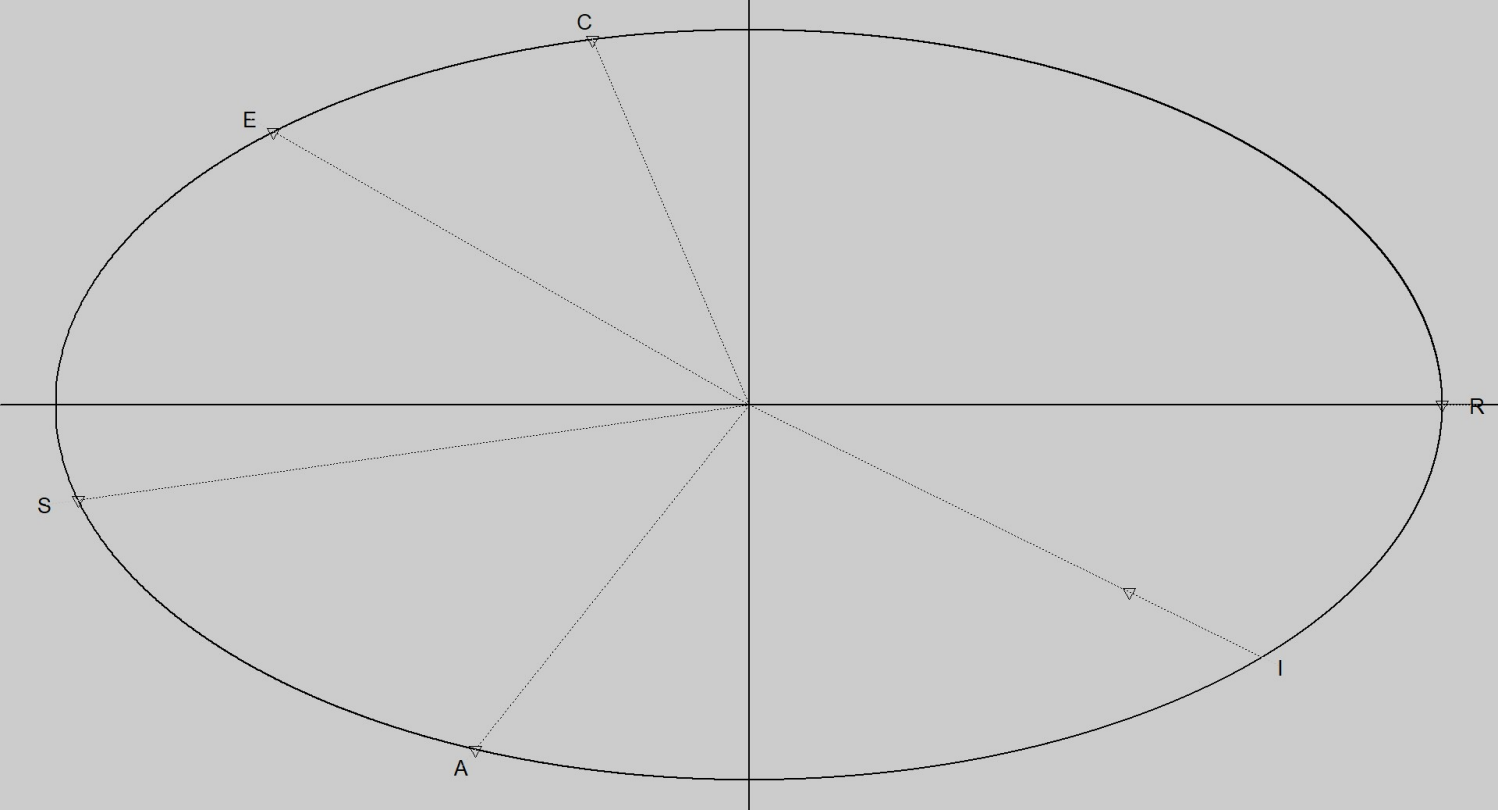
Verified study specific circumplex structure of the RIASEC model. R = realistic dimension, I = investigative dimension, A = artistic dimension, S = social dimension, E = enterprising dimension, C = conventional dimension.

The SIMON-I questionnaire (see also S1 Table in Supporting Information) was presented to all students to measure the six RIASEC dimensions of the Holland model as described in the introduction [12]. Derived from the specific item scores, all student participants received a RIASEC profile, with each dimension scoring between 0 (low) and 100 (high). For the present study, the RIASEC scales showed a reliability of .92, .87, .91, .92, .93 and .90 respectively, measured through a Cronbach’s alpha [34]. To confirm the validity of the RIASEC model for the present study’s data, we performed a confirmatory factor analysis on the circular structure of the RIASEC dimensions using the CirCe package in R [35–36]. Fig 1 shows the resulting circular structure for the present data sample. Confirmatory factor analysis (CFA) included several measures of model fit (*Standardized Root Mean square Residual* = 0.051; *Normed Fit Index* = 0.97; *Comparative Fit Index* = 0.97; *Goodness of Fit Index* = 0.99), all pointing towards a good to excellent circular fit. For an overview on interpretation of these indices, we refer to the exhaustive listings provided by Kenny [37]. Additionally, we also verified the circular RIASEC order and structure by conducting a *randomization test of hypothesized order relations* (RTOR) using the RANDALL package [38–40]. Results of this RTOR analysis revealed a *correspondence index* of .92, while a circular fit of the data also reached significance, *p* = .02. For a full discussion on the RANDALL function and RTOR analyses, we refer to [38–40]. In sum, the theorized circular fit for our RIASEC data was confirmed by both CFA and RTOR analyses.

#### Student PE interest fit and study program interest diversity

Next, the student PE fit between the vocational interest of the student and her/his study program was established using the correlational fit measure. To calculate the correlational fit measure (shape resemblance between the hexagonal pattern of student and study program profile) for each student, each student’s RIASEC profile was correlated with his or her study program RIASEC profile based on the profiles of successful and persistent students. As this correlational fit measure also represents a deviance, the measure (on a scale of -1 to 1) was then rescaled to an easy-to-interpret interest deviance between 0 and 1, with D COR = 1 - (correlational fit + 1) / 2. As an example, a student with a correlational fit measurement of 0.76 would rescale to D COR = 0.12, indicating the student’s RIASEC profile deviates 12% in regard to the profile of his or her study program.

A program interest diversity measure should indicate how deviant students’ interests are in a given study program of choice. By averaging out D COR across students in a program, we can obtain a measure of interest diversity AD COR for each study program, that represent how much students diverge on average from the program profile. As an example, a study program with an AD COR of 0.23 indicates that the RIASEC profiles of students within the program deviate (on average) 23% in regard to the program profile.

#### Autonomous and controlled motivation

The Self-Regulation Questionnaire was presented to all students to measure their autonomous (8 items) and controlled (8 items) motivation [41]. For the present study, the autonomous and controlled motivation subscales showed a reliability of .86 and .87 respectively, measured through a Cronbach’s alpha [34]. A factor analyses on both the autonomous and controlled motivation subscales revealed the expected two factor structure, explaining 52% of the variance. Items from the autonomous subscale showed high loadings on the autonomous factor (*M* = 0.70, ranging from 0.50 to 0.83) but not on the controlled factor. In contrast, items from the controlled subscale showed high loadings on the controlled factor (*M* = 0.73, ranging from 0.70 to 0.77).

#### Study results

GPA is a widely known and used measure of study success [42]. However, as Graham already pointed out, the validity and reliability of such a measure cannot be merely assumed, which forms a widespread problem in literature concerning study success [43]. Indeed, validity and reliability are function of both sample and measure, rendering GPA results from past samples insufficient [44]. To ensure reliability and validity of the GPA measure, not only towards research, but especially towards the eventual degree of students, the present study’s featuring open access university has installed several precautions embedded in the teaching and grading procedures for each study program. Considering the open access, it is important to note that all programs are strictly stratified in the first year. Because all students take the same courses, GPA is fully comparable within a program. For means of validity, attainment levels for all programs are actively protected by national and regional law. In other words, what students need to know in theory and practice is officially decreed and controlled by the government. For means of reliability, each program consists of 60 study points, divided over several courses (about ten courses for each program), taught by different professors and lectors to avoid rater bias. The exams for each program’s course are spread across a number of (non) periodical methods including but not limited to written exams, multiple choice exams (usually corrected automatically by use of computer), oral exams and essay writing to avoid methodical bias. In case of failing an exam, a student always has the right to resit the exam and even an appeal if irregularities (like dubious questions) were established. Both the resit and the appeal actively counter low reliability of exams. Importantly, we have included a PASS measure in the present study. In order to earn a PASS for his or her first year (and all other years), a student has to pass all courses of a study program by obtaining a course score of at least 10/20 for each course. This passing measure is an excellent countermeasure against possible overly optimistic exam marking in some courses of a program, as a student has to pass all courses of that program to obtain a PASS. Indeed, if a student earns a PASS, the results from all courses unanimously indicate the student has mastered the learning material for the first year. On the other hand, if there are courses in a program that are too difficult in comparison to the other courses, to the extent that no one would succeed the program, the discrepancy between the PASS rate (which would be zero) and the average GPA of the program should alert us to problematic (too strict) grading for those specific courses. For these specific reasons, we have included both PASS and GPA as measures of study results. GPA indicates the global result of the student in his first year study program on a scale from 0 to 1,000. PASS was assessed dichotomously (1/0) at the first year student level, and averaged out at the program level as a PASS rate, across students. If these study result measures are reliable and valid, both measures should correlate highly and should display similar results at the program level.

Despite these precautions, we still deem it possible (although unlikely) that a student’s GPA could be biased due to specific program choice and subsequent deviant grading. As such, we have taken a conservative approach by obtaining an estimate of the maximal hypothetical bias for GPA by calculating the intra class correlation coefficient through a multilevel model. For our purposes, this coefficient indicates how much of the variance in individual GPA can be attributed to the program level. Practically, the coefficient provides us with an estimate of the maximum bias due to possible different grading standards in different programs. However, stronger students could also systematically choose more difficult programs. This confound cannot be disentangled within the scope of the present study. As a results, we have included the intra class correlation coefficient as an absolute maximum of hypothetical bias in a student’s GPA due to the program of his/her choice.

### Analyses

To avoid unwanted bias of (potentially) skewed distributions of the correlational data on outcomes and subsequent conclusions, we performed all statistical testing on standardized scores by taking the z—scores of the D COR measures (by subtracting the grand mean and dividing by the grand standard deviation of the full data set) as the base measure. Averaging out these z—scores over programs renders the standardized equivalent of the AD COR measures.

To address our first research question, we inspected the variance of interest diversity over programs. The interest diversity of open access programs was tested against our control group that had to pass an entry exam. We also regress interest diversity (AD COR) on autonomous and controlled motivation to test whether both types of motivation in the student population are indeed linked to the variance in program interest diversity. To address our second research question regarding the influence of interest diversity on study results, we have regressed the average study program GPA and PASS on program interest diversity. As we are pitting three hypotheses against each other, we have considered both linear and curvilinear relations. To address our final research question regarding the comparison between the individual PE fit effect and the environmental program interest diversity effect, we have constructed two hierarchical models, a linear one for GPA and a logistic one for PASS In these models, we have investigated the effect of individual PE fit on study results, the effect of program interest diversity on study results and the cross-level interaction between individual PE fit and study program diversity. All analyses were conducted using R(Studio), SPSS and HLM software.

## Results

Table 2 shows the program summary containing the reference number, the scores on the R, I, A, S, E and C dimensions, the average student GPA, the student PASS rates, the AD COR interest diversity, the scores on autonomous (AMOT) and controlled (CMOT) motivation, the response rate (RR) on the SIMON-I questionnaire and finally the number of students (N). The correlation of GPA and PASS rates amounted to 0.80. Closer inspection revealed there was one program, *Geology* (36) with 0% PASS rate. Because this find could prove problematic, we considered the average GPA (370.29) of the program, which was very low (compared to the other programs). The correlation between GPA and PASS did not change by excluding Geology (36). There does not seem to be a huge discrepancy between the GPA and PASS results for Geology (36). Because 0% PASS rate is still a huge outlier, we decided to act conservatively and pay special attention to the Geology (36) result in specific PASS analyses.

**Table 2:** Descriptive statistics for all study programs.

Number	R	I	A	S	E	C	GPA	PASS	AD COR	AMOT	CMOT	N	RR
1	6.36	32.06	35.97	58.17	27.10	10.45	521.28	0.49	0.12	15.61	7.94	424	0.82
2	8.52	22.68	56.67	36.72	55.67	14.97	414.40	0.31	0.11	14.7	8.06	121	0.95
3	19.65	37.31	15.89	14.31	18.51	27.47	512.23	0.50	0.26	15.27	8.35	26	0.67
4	4.08	24.21	33.78	74.95	25.05	13.76	560.56	0.67	0.06	15.16	8.20	106	0.88
5	9.50	25.53	38.85	37.69	53.31	20.15	418.59	0.34	0.18	15.02	8.48	86	0.90
6	7.56	25.10	32.02	37.93	55.87	35.51	407.08	0.27	0.16	15.39	8.13	298	0.61
7	6.15	30.46	42.92	50.85	37.97	11.83	390.17	0.23	0.13	15.99	8.37	35	0.85
8	9.87	27.75	27.88	46.99	30.12	19.19	461.54	0.33	0.21	15.1	8.21	149	0.60
9	6.13	34.34	37.18	65.38	23.28	12.43	633.17	0.60	0.09	15.09	8.70	60	0.91
10	8.18	29.01	20.26	37.59	25.12	13.46	436.94	0.35	0.22	14.84	8.00	49	0.70
11	7.13	34.41	56.02	45.76	31.17	17.36	408.00	0.50	0.13	14.75	7.00	6	0.29
12	6.86	22.71	59.15	38.71	21.47	7.43	484.55	0.45	0.10	15.39	8.77	161	0.83
13	4.97	24.24	57.69	51.44	29.44	7.51	626.00	0.71	0.06	16.64	6.86	7	0.41
14	11.66	28.55	49.22	31.18	28.81	12.94	453.04	0.33	0.20	15.59	8.26	75	0.64
15	6.25	20.00	43.34	33.79	19.57	8.48	356.73	0.18	0.14	14.42	8.89	33	0.45
16	8.73	41.63	60.18	52.38	27.20	6.55	372.50	0.50	0.12	16.5	7.83	6	0.30
17	10.32	24.33	68.82	34.06	20.10	5.70	339.78	0.19	0.08	16.55	7.90	36	0.49
18	28.71	43.49	43.64	17.83	15.25	10.27	435.29	0.29	0.16	14.26	8.24	17	0.53
19	7.32	31.56	56.32	61.76	25.00	7.97	577.17	0.83	0.05	17.06	5.42	6	0.46
20	18.08	45.68	22.78	31.77	18.06	13.69	428.40	0.34	0.18	15.49	7.36	179	0.66
21	13.79	40.32	21.61	46.63	17.87	11.62	430.13	0.27	0.15	14.81	7.77	361	0.75
22	15.17	49.44	23.51	36.26	20.64	17.74	524.65	0.49	0.17	15.38	8.35	210	0.78
23	32.60	59.13	20.36	24.49	27.10	20.09	479.13	0.41	0.18	15.35	8.93	202	0.81
24	22.03	25.20	21.19	22.84	66.53	54.16	524.12	0.45	0.12	14.74	8.96	360	0.69
25	16.03	54.35	26.30	34.54	20.76	14.99	503.03	0.39	0.14	15.25	7.98	129	0.78
26	46.44	30.82	56.25	20.56	31.20	15.70	486.63	0.48	0.18	15.06	7.86	62	0.43
27	51.95	40.67	22.22	13.61	32.86	22.57	539.51	0.52	0.19	15.19	8.70	222	0.65
28	14.10	16.84	23.73	27.22	66.83	47.84	476.72	0.39	0.12	14.12	8.96	330	0.61
29	33.53	45.37	19.20	20.84	22.78	19.14	524.52	0.45	0.23	13.93	9.28	75	0.82
30	59.70	32.15	22.71	13.39	26.77	19.42	421.68	0.29	0.18	14.09	8.20	349	0.84
31	6.22	16.44	47.33	35.99	30.01	10.94	433.15	0.33	0.14	14.83	8.75	133	0.67
32	17.12	54.26	22.00	22.97	13.05	11.32	463.81	0.37	0.13	14.9	8.12	67	0.66
33	23.24	50.75	28.77	27.18	13.18	6.94	390.70	0.36	0.12	15.06	7.12	50	0.68
34	22.94	47.74	17.11	21.77	18.10	17.70	479.05	0.39	0.18	14.61	7.53	38	0.66
35	33.58	46.81	23.97	11.56	15.11	11.03	400.60	0.40	0.15	15.47	7.71	47	0.68
36	33.90	46.25	31.00	20.11	15.59	10.42	370.29	0.00	0.17	14.54	8.48	14	0.67
37	38.45	39.52	20.57	29.66	13.64	15.69	522.15	0.46	0.26	14.88	10.79	13	0.59
38	31.50	26.76	24.90	7.58	20.09	15.64	459.51	0.51	0.24	14.44	7.73	43	0.66
39	8.74	18.79	25.18	42.15	71.35	42.78	476.58	0.44	0.11	15.01	8.39	50	0.70
*	17.51	47.68	23.41	44.68	26.40	14.81	702.37	0.93	0.17	16.38	8.36	192	0.86

Note. Number: see Table 1, R = average program scores (on 100) on realistic dimension, I = average program scores (on 100) on investigative dimension, A = average program scores (on 100) on artistic dimension, S = average program scores (on 100) on social dimension, E = average program scores (on 100) on enterprising dimension, C = average program scores (on 100) on conventional dimension, GPA = average program grade point average, PASS = average program pass-rate, AD COR = program interest diversity, AMOT = average program autonomous motivation, CMOT = average controlled program motivation, N = number of students in the program, RR = program response rate.

Important to note, the intra class correlation coefficient for GPA amounted to 6%. This means that only 6% of the variance in GPA can be attributed to the program level. In other words, the individual GPA is determined for 94% based on personal achievement. As such, the maximum possible bias of GPA due to non-equivalent grading can be estimated at 6%.

### Research question 1: Interest diversity of study programs

**Fig 2:**
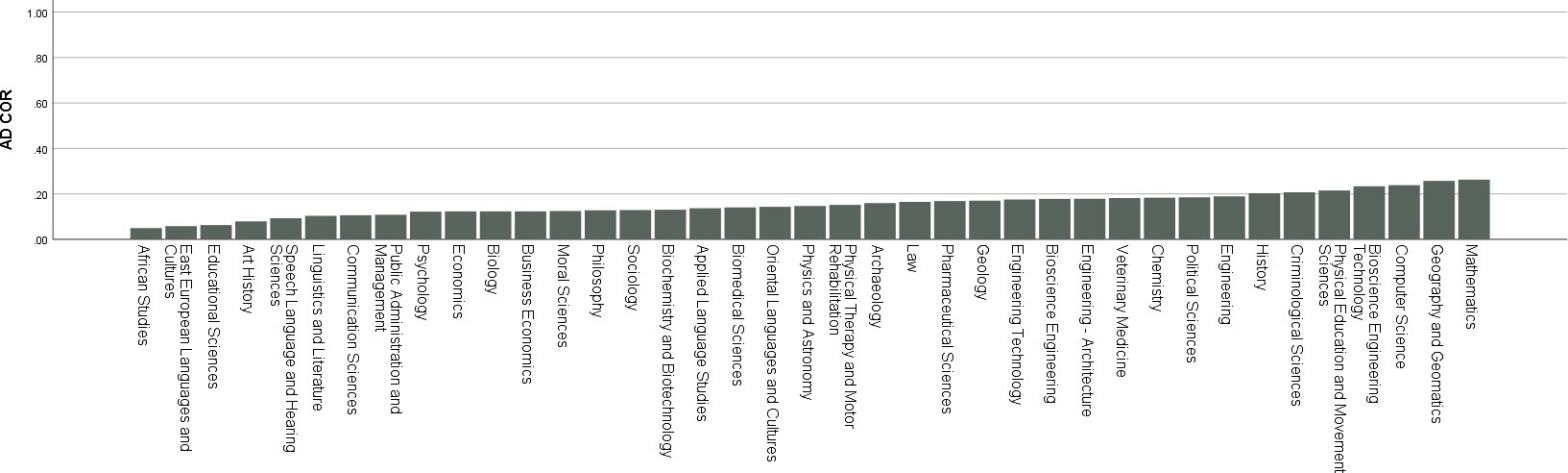
Interest diversity program spread across the 0–1 continuum. The X-axis displays the 39 study programs featuring in this study. Y-axis displays the program interest diversity expressed through an AD COR value.

Fig 2 shows the spread of interest diversity (AD COR) across programs (*M* = .15 ; *SD* = .05). This interest diversity also corresponds to a rather impressive average PE fit correlation of .70 between the RIASEC profiles of students and their program of choice. We thus clearly observe a concentration of programs at the lower end of the diversity continuum (due to the high average PE fit), ranging from .05 (African Studies) to .26 (Mathematics). This concentration at the lower end also means that 74% of the interest diversity continuum at the higher end remains unused. To formally test if an open access environment indeed results in more interest diversity regarding study programs, we compared the interest diversity of 39 study programs with open access to the control group with restricted access. A two-sided one sample t-test indeed revealed a significant difference, *t*(38) = -4.27, *p* < .001, with a somewhat large effect size of Cohen’s *d* = 0.69. We therefore conclude that the evidence shown confirms our hypothesis. Study programs in an open access environment indeed display a low interest diversity regarding their student population.

**Fig 3:**
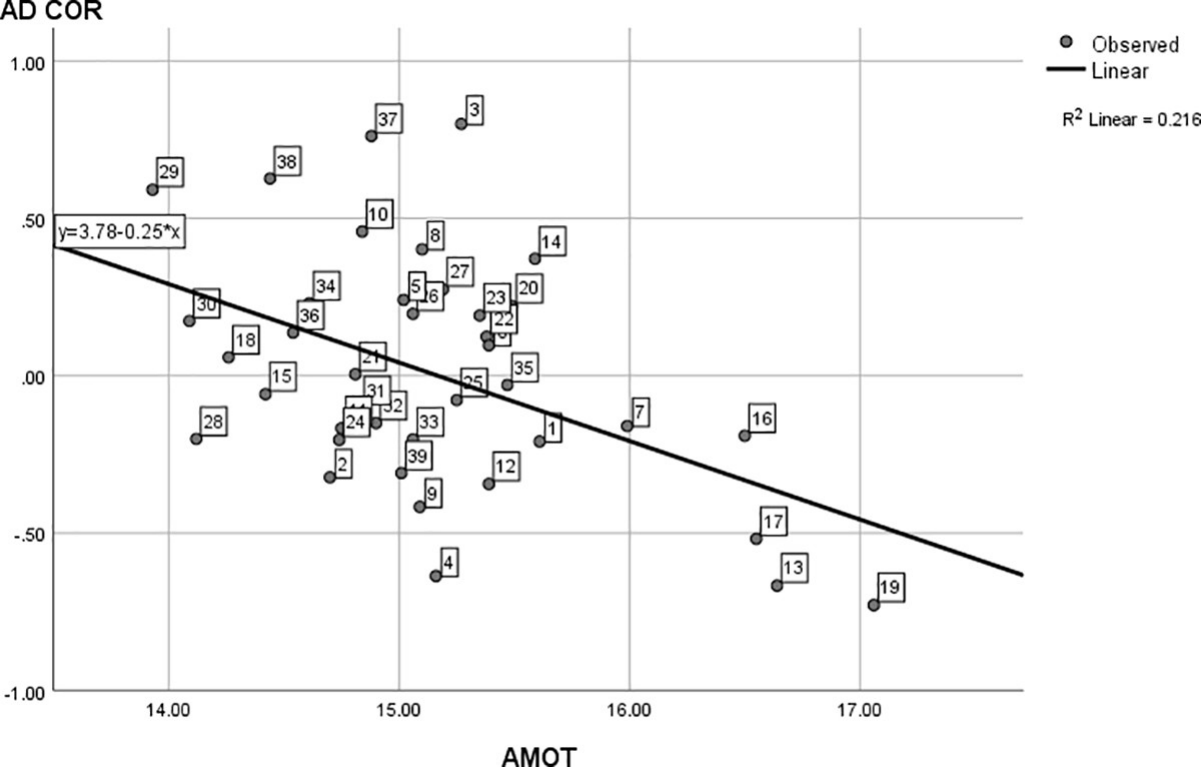
The linear regression of interest diversity on autonomous motivation. Interest Diversity is measured using AD COR (based on D COR z–scores), with AMOT = autonomous motivation. The data points (dotted study programs) are labeled analogous to Table 1.

**Fig 4:**
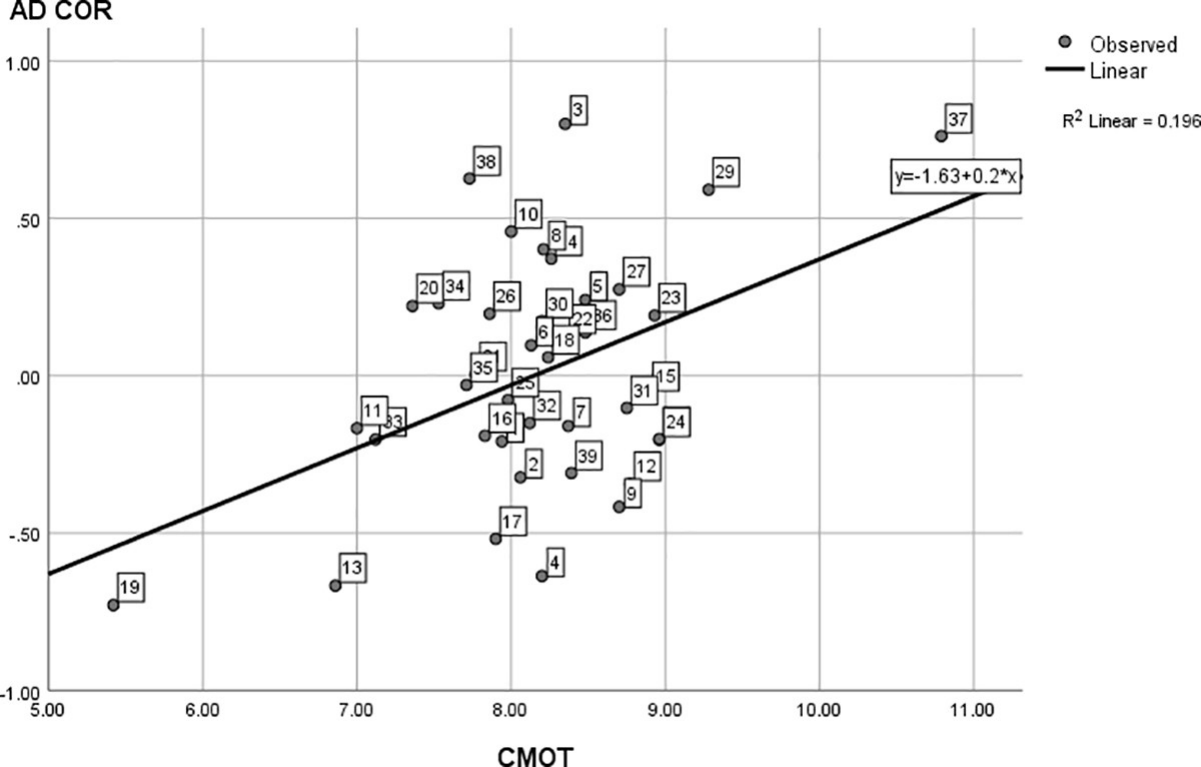
The linear regression of interest diversity on controlled motivation. Interest Diversity is measured using AD COR (based on D COR z–scores), with CMOT = controlled motivation. The data points (dotted study programs) are labeled analogous to Table 1.

For the second part of our first research question, Fig 2 also shows interest diversity does still display quite some variance across programs at the lower end of the continuum. For instance, the interest diversity in Mathematics is about five times larger than the diversity in African studies. To test whether this variance is linked to motivation, we regressed interest diversity (AD COR) on (autonomous and controlled) motivation. The omnibus test proved to be significant, *F*(2, 36) = 6.86, *p* = .003, with an explained variance of 28%. Standardized regression coefficients (*β*_1_ = -0.33, *β*_2_ = 0.28, for autonomous and controlled motivation respectively) indicate a negative relation between program interest diversity and average autonomous motivation in the program student population, and a positive relation between interest diversity and average controlled motivation in the program student population. Closer graphical inspection (Figs 3 and 4) of the individual effects also reveal the effects are especially present at (relatively) very high and very low levels of controlled and autonomous motivation. These findings confirm our hypothesis that program interest diversity is indeed related to motivation in an open access environment. Programs with low interest diversity have a student population with high autonomous motivation and vice versa. Programs with high diversity are linked to student populations with a higher controlled motivation while programs with low diversity are linked to populations with a lower controlled motivation.

### Research question 2: Effects of interest program diversity

**Fig 5:**
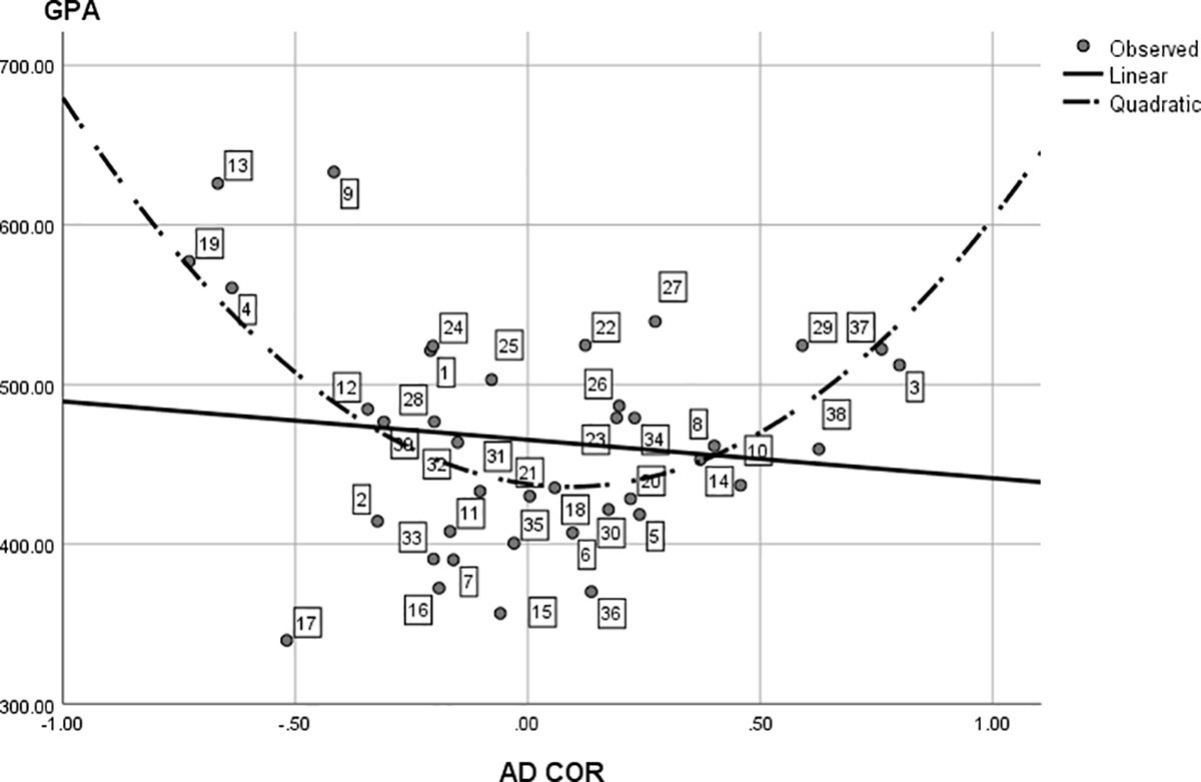
The (curvi-) linear regression of study program average results (GPA) on study program interest diversity. Interest Diversity is measured using AD COR (based on D COR z–scores), with GPA = average grade point average for each study program. The linear regression is depicted as a full line, the quadratic regression is depicted as an interrupted line. The data points (dotted study programs) are labeled analogous to Table 1.

**Fig 6:**
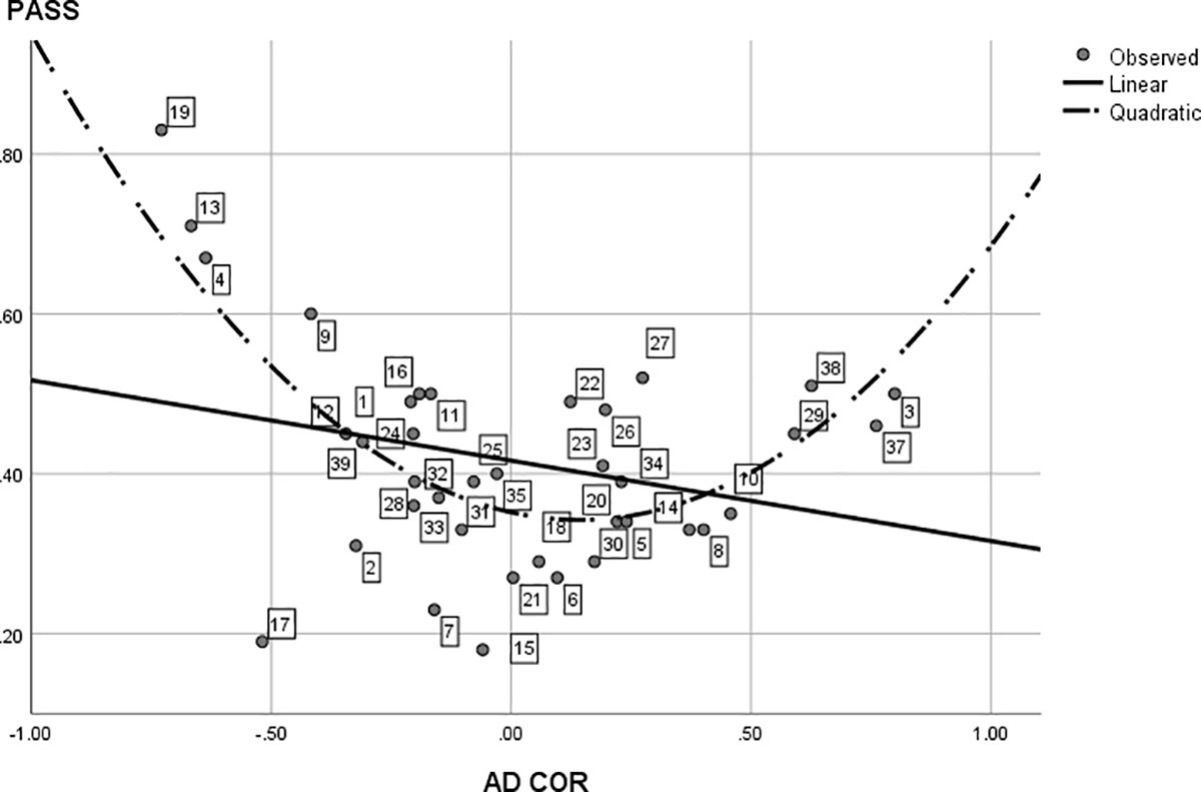
The (curvi-) linear regression of average study program results (PASS) on study program interest diversity. Interest Diversity is measured using AD COR (based on D COR z–scores), with PASS = average pass rate for each study program. The linear regression is depicted as a full line, the quadratic regression is depicted as an interrupted line. The data points (dotted study programs) are labeled analogous to Table 1.With a pass rate of 0%, Geology (36) was considered an outlier and was removed from analyses.

To examine the possible direct effect of interest diversity in study programs on average program study results, we conducted two (curvi-) linear regressions of both average program GPA and PASS rates on interest diversity (AD COR). Figs 5 and 6 show the regressions of average program study results on program interest diversity. We obtained similar results for both measures of study results. Indeed, the linear regressions of study results on interest diversity were not significant *F*(1,37) = 0.63, *p* = .43 and *F*(1,36) = 3.01, *p* = .09 for GPA and PASS respectively. The curvilinear regressions of study results on interest diversity however, were significant with high levels of explained variance, *F*(2,36) = 7.19, *p* = .002, *R*^2^ = .29 and *F*(2,35) = 13.84, *p* < .001, *R*^2^ = .44 for GPA and PASS respectively. Adding the Geology (36) results to the PASS regression rendered similar results. These curvilinear findings provide evidence that rising program interest diversity has a mixed effect on the average study results of the program’s student population. Different programs do seem to reward different interest patterns (Smart, 2000). More specifically, a very low diversity in a small number of programs is associated with very high average study results. However, for the majority of the programs that had a higher diversity to begin with, we observe that study results tend to improve as program interest diversity rises.

As we were curious about the nature of these curvilinear effects, we ran further post hoc analyses. Closer inspection of Figs 5 and 6 revealed that the left, descending part of the curvilinear relation is largely caused by the study results and interest diversity of four programs: *Educational Sciences*, *Speech Language and Hearing Sciences*, *East European Languages and Cultures* and *African Studies*. We decided to compare the six dimension RIASEC profiles of these four programs to each other and to the other programs. Table 3 shows the correlation of the four RIASEC program patterns. The high correlations indicate these programs have very similar interest patterns. When comparing the RIASEC interest profile of these four programs to the other programs in the present study, these specific programs have relatively very high scores on the social S dimension (rankings 1, 2, 3 and 6 out of 39), and very low scores on the realistic R dimension (rankings 30, 37, 38 and 39 out of 39). For these specific programs, a low interest diversity is tied to better study results, as is shown by the correlation between their interest diversity and their average study results: AD COR correlates -.47 with GPA and -.88 with PASS.

**Table 3:** Comparison (correlations) RIASEC profiles of *educational sciences*, *speech language and hearing sciences*, *East European Languages and Cultures* and *African Studies*.

Number	Program	4	9	13	19
4	Educational Sciences	—	.97	.79	.87
9	Speech Language and Hearing Sciences		—	.83	.93
13	East European Languages and Cultures			—	.97
19	African Studies				—

**Fig 7:**
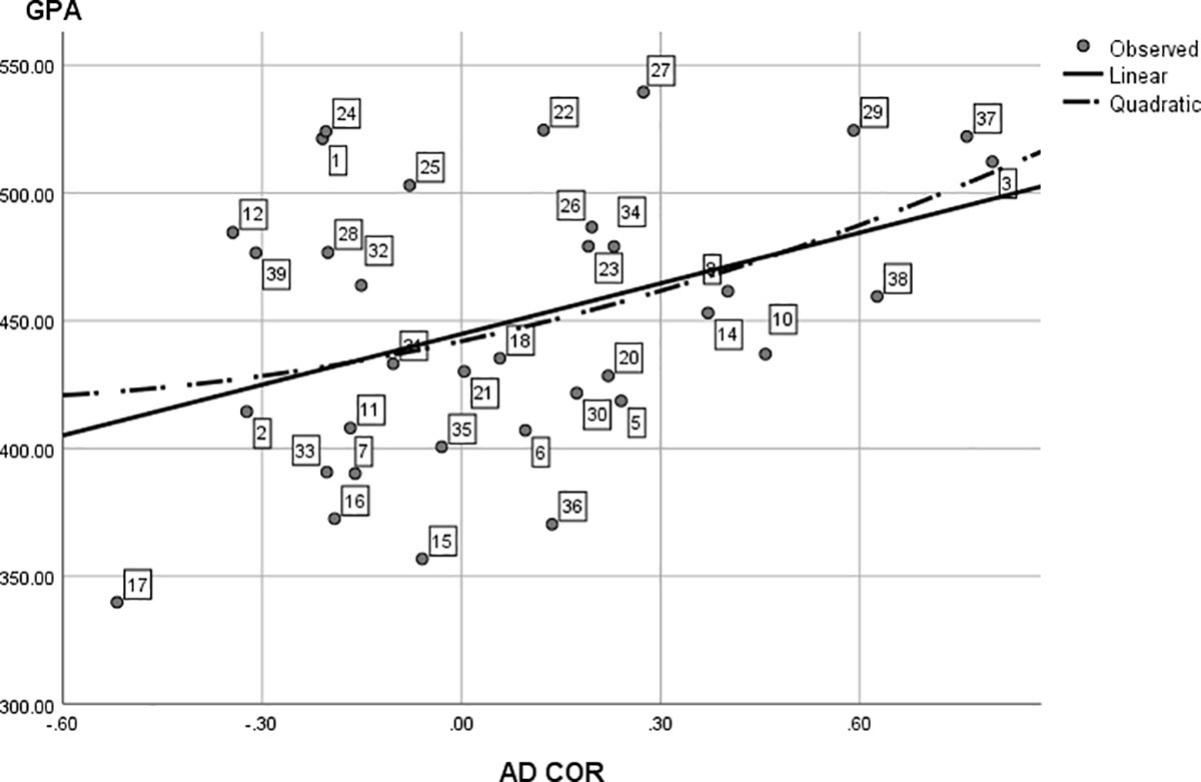
Post hoc analysis: The (Curvi-) linear regression of average study program results (GPA) on study program interest diversity. The data points (study programs) are labeled analogous to Table 1 and are identical to Fig 4, with omission of points 4, 9, 13 and 19 that were discussed separately.

When considering the other programs of the present study, we repeated the regression of study results (GPA and PASS) on interest diversity by excluding the results from these four programs. Fig 7 now clearly shows a linear regression best explains the relation between program interest diversity and average study results compared to a curvilinear one. Statistically, the linear regression of GPA on interest diversity reached significance, *F*(1, 33) = 6.13, *p* = .02, *R*^2^ = .16, while the curvilinear one no longer did, *F*(2, 32) = 3.10, *p* = .06.

**Fig 8:**
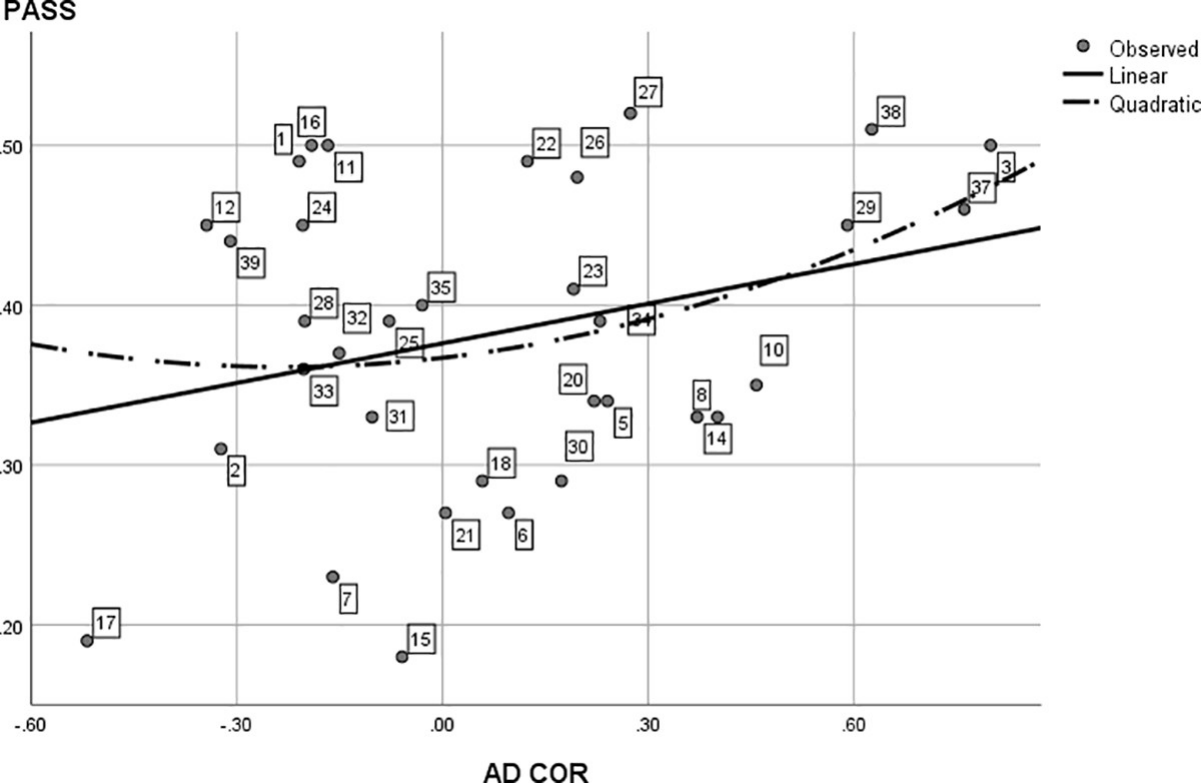
Post hoc Analysis: The (curvi)linear regression of average study program results (PASS) on study program interest diversity. The data points (study programs) are labeled analogous to Table 1 and are identical to Fig 5, with omission of points 4, 9, 13 and 19 that were discussed separately. With a pass rate of 0%, Geology (36) was considered an outlier and was removed from analyses.

Fig 8 shows a somewhat similar pattern. Although the linear regression of PASS on interest diversity did not reach significance, *F*(1, 32) = 2.84, *p* = .10, interest diversity still showed a strong linear trend towards an effect by explaining 8% of the variance in program study results. The curvilinear regression did not reach significance, *F*(2, 31) = 1.72, *p* = .20. The addition of Geology (36) to the regression rendered similar results, although the explained linear variance only amounted to 5%.

In sum, these results confirm our hypothesis that interest diversity has a direct, but mixed effect on average study program results in an open access environment. Different programs seem to reward different interest patterns. In general, a higher interest diversity of the student population has a positive effect on average program study results. However, this effect seems to reverse for very specific programs displaying a very high social dimension and a very low realistic dimension. These programs reach high average study results if the student population shows very low levels of interest diversity.

### Research question 3: Individual student PE fit and program interest diversity

To compare the effect of program interest diversity on study results to the effect of individual PE fit, we have performed multilevel analyses of student (level 1) and program (level 2) effects on study results. As GPA is a linear variable and PASS is a dichotomous variable, we constructed a linear and a logistic multilevel model. Effect sizes for the different effects at the individual level, program level or both levels combined (full model) were calculated by combining the variance components (GPA model) and model deviance statistics (PASS model) into a pseudo *R*^2^.

Table 4 shows the final version of the GPA model (see also [45] for a discussion on multiplicative interaction analysis). Though significant, the fit of the individual student with this study environment (measured through the D COR measure) only explained 0.6% of the variance in study results at the specific individual level and the general full GPA model. In contrast, program interest diversity (AD COR) explained 17% of the variance in average study results at the program level and 1% of the variance in the full model.

**Table 4:** Multilevel GPA model.

**A**			* *		* *
Multilevel GPA Model					
Level-1 Model					
*GPA*_*ij*_ *= β*_*0j*_ *+ β*_1j_*D COR_*ij*_ *+ r*_*ij*_					
Level-2 Model					
*β*_*0j*_ *= γ*_*00*_ *+ γ*_01_*AD COR_*j*_ *+ γ*_02_*AD COR_*j*_^*2*^ *+ u*_*0j*_					
*β*_*1j*_ *= γ*_*10*_ *+ u*_*1j*_					
Mixed Model					
*GPA*_*ij*_ *= γ*_*00*_ *+ γ*_01_* AD COR_*j*_ *+ γ*_02_* AD COR_*j*_^*2*^					
*+ γ*_10_*D COR_*ij*_					
*+ u*_*0j*_ *+ r*_*ij*_					
**B**			* *		* *
Final estimation of fixed effects	Coefficient	Standard Error	*t*-ratio	appr. *d*.*f*.	*p*-value
Fixed Effect					
For INTRCPT1, *β*_*0*_					
INTRCPT2, *γ*_*00*_	446.22	10.12	44.07	36	< .001
*AD COR*, *γ*_*01*_	-28.73	31.63	-0.91	36	0.37
*AD COR*^*2*^, *γ*_*02*_	178.33	55.06	3.24	36	0.003
For *D COR* slope, *β*_*1*_					
INTRCPT2, *γ*_*10*_	-18.03	3.54	-5.09	4595	< .001
**C**			* *		* *
Final estimation of variance components	Standard Deviation	Variance Component	*d*.*f*.	*χ*2	*p*-value
Random Effect					
INTRCPT1, *u*_*0*_	48.66	2367.93	36	259.14	< .001
level-1, *r*	213.16	45440.33	—	—	—

Note. GPA = grade point average, D COR = student PE interest fit, AD COR = program interest diversity, appr. *d*. *f*. = approximated degrees of freedom. Model construction was conducted as follows. We first determined the amount of variance in study results (GPA) generated through both levels by using the intercept-only model. Level 1 (individual level) generated 94%, while level 2 (program level) generated the remaining 6% of variance in study results measured through GPA. Explained variance of the different effects was calculated using comparisons to the intercept-only model. Before making the actual models, we tested the possible influence of study program SIMON-I response rate and population (number of students) by adding them to the intercept model. Both tests were not significant, *t*(36) = 1.14, *p* = .26 and *t*(36) = 0.41, *p* = .69 respectively. Both group variables were thus removed from the intercept model. The D COR predictor was added to the model as a program centered variable, removing the variance between programs. This variance between programs was then added through the curvilinear effect of AD COR from research question 2 completing the model. Note that the linear term AD COR renders a non-significant result, while the quadratic term did reach significance. Though the absence or presence of the linear term does not change the curvilinear nature of the interest diversity effect, we have decided to keep the linear term as a part of the model due to the multiplicative interaction between the student and the program level.

As hypothesized regarding our third question, our GPA multilevel model thus indicates the influence of the individual’s PE fit on individual study results can indeed be considered low. However, at the same time, these GPA model results also reveal that interest diversity over programs explains much more variance in average study results in that program compared to the individual level.

We also tested the addition of a cross-level interaction (through a random slope for PE fit) between interest diversity (AD COR) at the program level and PE-fit (D COR) at the individual level. The chi-squared test returned a non-significant result, *χ*^2^ (38) = 45.26, *p* = 0.20. This result indicates the individual PE fit does not interact with the program interest diversity. In other words, there is no different individual effect of PE fit on study results depending on the study program environment.

Table 5 shows the final PASS model. The results are largely analogous to those from the GPA model. The deviance statistic from the full models (containing predictors) compared to the intercept only model revealed that the predictor models were significant. However, the models showed very low pseudo *R*^2^ (around 0.1%), for both individual PE fit and program interest diversity when considering full model deviance (all levels combined). PE fit only reached a pseudo *R*^2^ of about 0.1% at the individual level, while the pseudo *R*^2^ for interest diversity did reach 37% at the program level.

**Table 5:** Multilevel PASS model.

**A**			* *		* *
Multilevel PASS model					
Level-1 Model					
*Prob (PASS*_*ij*_ *= 1|βj) = ϕ*_*ij*_					
*log[ϕ*_*ij*_*/(1 - ϕ*_*ij*_*)] = η*_*ij*_					
*η*_*ij*_ *= β0*_*j*_ *+ β1*_j_*(D COR_*ij*_*)*					
Level-2 Model					
*β*_*0j*_ *= γ*_*00*_ *+ γ*_01_*AD COR_*j*_ *+ γ*_02_*AD COR_*j*_^*2*^ *+ u*_*0j*_					
*β*_*1j*_ *= γ*_*10*_ *+ u*_*1j*_					
Mixed Model					
*η*_*ij*_ *= γ*_*00*_ *+ γ*_01_* AD COR_*j*_ *+ γ*_02_* AD COR_*j*_*^2^*					
*+ γ*_10_*D COR_*ij*_					
*+ u*_*0j*_					
**B**			* *		* *
Final estimation of fixed effects	Coefficient	Standard Error	*t*-ratio	appr. *d*.*f*.	*p*-value
Fixed Effect					
For INTRCPT1, *β*_*0*_					
INTRCPT2, *γ*_*00*_	-0.38	0.07	-5.44	36	< .001
*AD COR*, *γ*_*01*_	-0.4	0.23	-1.715	36	0.1
*AD COR^2^*, *γ*_*02*_	1.91	0.48	4.02	36	< .001
For *D COR* slope, *β*_*1*_					
INTRCPT2, *γ*_*10*_	-0.15	0.04	-4.1	4595	< .001
**C**			* *		* *
Final estimation of variance components	Standard Deviation	Variance Component	*d*.*f*.	*χ*2	*p*-value
Random Effect					
INTRCPT1, *u*_*0*_	0.33	0.11	36	157.01	< .001
Model Deviance = 14571–5 estimated parameters	—	—	—	—	—

Note. GPA = grade point average, D COR = student PE interest fit, AD COR = program interest diversity, appr. *d*. *f*. = approximated degrees of freedom. Model construction: analogous to the multilevel GPA model. Program SIMON-I response rate and population again tested non-significant, *t*(36) = 0.60, *p* = .55 and *t*(36) = -0.46, *p* = .65 and were removed from the model.

As hypothesized regarding our third question, our PASS multilevel model thus indicates the influence of the individual’s PE fit on individual study results can indeed be considered low. In contrast to the GPA model, both individual PE fit and program interest diversity displayed very low levels of explained variance when considering full model deviance. Analogous to the GPA model, PASS model results also reveal that interest diversity in the program explains much more variance in typical passing rates for that program, across students.

The addition of a random slope for the PE predictor again resulted in a non-significant result, *χ*^2^ (38) = 35.04, *p* > .50. Hence, we do not find any evidence for a cross-level interaction between individual student PE fit and program interest diversity on study results.

In sum, these findings again confirm our hypothesis that the individual effect of PE fit on study results is small to almost non-existent in an open access environment, while interest diversity has a profound effect on results at the program level. For the full models, we have found mixed evidence that program interest diversity is more explanative towards study results than individual PE fit. Furthermore, we did not find any evidence for an interaction effect between group level program interest diversity on individual study results.

## Discussion

Vocational interest refers to the liking or disliking of certain activities or environments, represented by a number of base dimensions and characterized by the properties of prediction, contextualization, stability and motivation [7,15,8,3,4,11,5,13,6]. Literature has shown that individual person-environment fit (PE fit) between students and study programs influences higher education study results [21,1,24,22,26,23,9,25]. These studies however, mostly focus on the student, while leaving the study environment underdeveloped [2]. As such, we are uncertain how diverse study programs actually are in terms of vocational interest of their student populations. Moreover, we also do not know whether and how program interest diversity exerts an influence on study outcomes like grade point average (GPA). Finally, when studying theoretical concepts like interest diversity or PE fit, admission restrictions to study programs in higher education, like entry exams or GPA requirements, may imply a selection bias in student intake that may influence the effects of PE fit.

The present prospective study set out to remedy these voids in literature. As such, the present study was conducted in an open access environment, exploring the interactions between individual interest PE fit and environment program diversity. Although we derived our hypotheses from homogeneity theory, we do not consider interest diversity as a mere synonym for homogeneity [27,28,20]. In fact, our operationalization of the interest diversity construct is quite unique, as it reuses measures of PE fit as an indication of how a student deviates from his study program profile. By averaging out these deviances across a program, a continuous measure of program interest diversity was obtained, through the use of a very large sample of students. Using this interest diversity measure, we assessed three research questions. We investigated these questions in a population of bachelor students starting their academic trajectory at a large Western-European university (Shanghai top 100) across eleven faculties with an open access policy.

During the present study, special care was given to the validity and reliability of predictors and study result measurement. As indicated by Graham and Harris, reliability and validity are function of both sample and measure [43,44]. The university where the study took place already had a number of measures in place to guard the reliability and validity of study results. Apart from the widely known GPA measure [42], we also added an extra measure of study success through the PASS rate. A student only received a PASS if he or she succeeded for all courses of the program. As explained in the method section, if our study result measures are reliable, both measures should show a high correlation and both measures should show the same result pattern. Our analyses indeed confirmed both predictions. Moreover, the intra class correlation coefficient for GPA amounted to only 6%. This find indicates that the bias in GPA due to non-equivalent quoting can only amount to a maximum of 6%. To which extent this percentage is determined by stronger students systematically choosing certain programs or rater-bias cannot be disentangled within the current study. Still, as a result, a student’s individual GPA is to a very large degree (at least 94%) determined by his personal achievement and not through the specifics of program followed. As such, we are convinced that we have taken the necessary precautions to ensure the reliability and validity of our study result measures and the overall results of the present study.

For our first research question, we investigated how diverse study programs actually are in the vocational interest of their student population. We hypothesized program interest diversity would be low, as predicted by the homogeneity assumption. We also expected that this general low interest diversity would still show variance over the range of programs, linked to the motivation of their student populations. Indeed, some programs are chosen through autonomous motivation by students who are highly interested in their program of choice. As students are highly interested in their program of choice, such programs should display a low interest diversity. Other programs could be more attractive to students who have ulterior motives like pleasing their parents and should display a higher interest diversity. As students are less intrinsically interested in their program of choice, these programs display more variance in student vocational interest, resulting in a higher interest diversity. Results for our first question indeed showed that program diversity was low across all study programs, leaving 74% of the higher end diversity continuum unused. On an individual level this find indicates that in general, the PE fit between students and their programs is quite high: RIASEC profiles between student and program correlate .70, on average. Indeed, students predominantly seem to choose a higher education study program that fits their interests quite well when given the opportunity, as is the case in an open access environment. Results also showed that the variance in program interest diversity is related to motivation. Study programs with low interest diversity were linked to students with relatively higher autonomous motivation, while programs with a higher interest diversity were linked to students with a higher controlled motivation. This relation between student motivation and interest diversity indicates that some programs do attract more students with a higher controlled motivation.

For our second research question, we explored the direct effect of the program interest diversity on average study results. As different programs could reward different interest patterns, we took a conservative approach and pitted three hypotheses against each other on how program interest diversity would influence average program study results. The curvilinear relation between program interest diversity and average study results provided evidence for our mixed effects hypothesis. Different programs indeed rewarded different interest patterns [29]. To provide an explanation for this curvilinear effect, we performed a post-hoc analysis. Results of this analysis showed that in general, larger program interest diversity was linked to better average study results. In other words, programs with more interest diversity in their student population showed better average results. However, some study programs with very specific interest patterns that scored high on the Social dimension and low on the Realistic dimension showed an opposite relation: lower program interest diversity in student populations in such environments was associated with better study results. To improve general study results, these findings suggest policy makers and institutions in (open access) higher education should allow for interest diversity in the student population of study programs. At the same time, policy should also ensure a sufficiently high individual student PE interest fit, as literature already suggested [21,1,24,22,26,23,9,25]. However, to ensure better study results for very specific programs (high on the social dimension and low on the practical dimension) like Educational Sciences, the fit between student and program should indeed be as high as possible, resulting in a (very) low program interest diversity and a very high individual PE interest fit. Finally, results also revealed criterion validity for our continuous approach of program interest diversity: up to 44% of the variance in average study program results can be explained by program interest diversity in student populations. As such, our continuous approach of interest diversity represents a valuable addition to the measurement of internal similarity of environments usually determined through dichotomous test statistics [30–32].

For our final research question we compared the effect of program interest diversity on study results to the effect of individual PE fit. We hypothesized that due to the low interest program diversity (or high internal similarity) the effect of PE fit would be low. We also tested the cross-level interaction of individual program fit and environmental program diversity. Analyses indicated that the effects at the individual level on study results were very modest at best: student PE fit only explained up to 0.6% at the individual level. Hence, in an open access higher education environment, the variance in PE fit between students and their program barely has a meaningful impact on individual study results. In opposition to these results at the individual level, program interest diversity explained up to 37% of the variance at program level, which is a huge contrast to the observed explained variance at the individual level. Moreover, program interest diversity of different study programs did not only influence average study results, we also obtained partial evidence that this diversity explained more study result variance in the total multilevel models than the individual indicators of PE fit. As only a small part of the total variance in study results (up to 6%) was situated at the program level to begin with, this is no small feat indeed. These findings are analogous to those found in our second question and provide additional evidence that higher education institutions should indeed consider program interest diversity when making policy decisions towards student orientation and admission. As a possible explanation, most students in this open system showed a high PE fit with their program of choice. In systems were choice is restricted (on the basis of exams or GPA requirements), students may have to choose for programs that match their interests less well, and then this (larger variety in) PE fit has a bigger impact on individual study results. Indeed, earlier research that examined PE fit effects on study results in constrained access systems typically observed more explained variance [22,10,26,23,24,9,25].

This discussion on the consequences of open access policy illustrates the importance of studying PE fit effects in a variety of study contexts. As Nauta already indicated, (study) environments remain understudied [2]. Entry exams or GPA requirements yield a selection bias in student intake that will influence the internal similarity in student populations, and therefore also the effects of the observed PE fit variance. Such contextual effect are likely partly responsible for the mixed results regarding the influence of PE fit on study results. For the first time in literature, the present study thus aimed at addressing this problem directly by conducting a PE fit/interest diversity study in a predefined open access environment, firmly rooted in existing theory regarding the possible influence of the environment. As theory predicted, the influence of the environment on outcomes in this open access set up becomes quite influential, while the individual level almost has no explanative power at all regarding study results. In other words, the open access environment causes study program interest diversity to have a profound influence on study results, while severely diminishing the influence of individual student PE fit.

To close the discussion on our third question, the variance in program interest diversity and student PE fit was limited to the extent the cross level interaction between individual and environment was not significant. In other words, program interest diversity did not influence the student PE fit-study results relation: effects of individual PE fit remained low, regardless of the interest diversity of study programs. These findings in our open access study environment are at odds with results from internal similarity research from Tracey and colleagues [9]. They showed that the effects of PE fit on individual study success was indeed constrained by the study environment. As an explanation, we speculate the open access system leads to such low interest variance that prevents a cross-level interaction between study program interest diversity and student PE fit.

### Limitations and future research

The present study is unique in its assessment of PE fit effects in an open access system. It would be interesting in the future to directly compare study environments with more constrained entry restrictions on the exact same measurements, using the exact same analyses. We speculate that such an approach would show enlarged PE fit effects in more restricted study programs, while the influence of interest diversity will diminish. The access restrictions could thus be a crucial factor in explaining the mixed findings in literature regarding PE fit, while elaborating literature with interest diversity research.

Our conceptualization of interest diversity and its motivational connection could also be used in organizational and occupational research. We predict that not all work environments will show the same amount of interest diversity. As access to the work environment works quite differently in comparison to access to higher education, we can also expect different effects. For instance, open access to certain jobs (low degree requirements) could result in higher interest diversity. Indeed, a student in an open access environment picks a certain program because that program is of particular interest to him or because his parents wants him to study that specific program. An employee could have other motives to pick a job. Employees who have no (or a low) degree can decide to work out of financial motives exclusively. Though highly speculative, we think that the different motivation in work and study contexts will lead to different patterns of interest diversity for both contexts and could ultimately end up explaining why the strength of the PE fit–study results relation is so underwhelming in comparison to the theoretical predictions.

## Conclusion

In the present study, we have assessed program interest diversity of student populations in study programs. In an open access environment, interest diversity of student populations in study programs is low. RIASEC profiles of students and programs correlate .70 on average. Interestingly, study programs with low interest diversity attract students with relatively higher autonomous motivation, while programs with a higher interest diversity show higher controlled motivation. Despite overall low diversity, the interest diversity of the program environment still had a profound effect on the program’s typical study results, while the influence of individual PE fit seems to be nonexistent or very limited at best. In order to enhance student study success, the present study has shown policy makers and educational institutions should focus on a sufficiently high PE fit amongst their student populations, while still allowing for some study program interest diversity.

## Supporting information

S1 Table SIMON-I questionnaire. (DOCX)
