# Supplementary material for: The effects of vocational interest on study results: Student person – environment fit and program interest diversity
Source: PLoS One. 2019 Apr 4;14(4):e0214618. doi: 10.1371/journal.pone.0214618 (PMC6448847; doi:10.1371/journal.pone.0214618)
Supplement: S1 Table — (DOCX) [file pone.0214618.s001.docx]

**Supporting information**

**S1 Table. SIMON-I Questionnaire.**

| **A** |  |  |  |  |
| --- | --- | --- | --- | --- |
| Activities | Dimension | YES | NO |  |
| Developing electronic systems | R |  |  |  |
| Analysing the grammatical structure of a sentence | I |  |  |  |
| Helping people with speech disorders | S |  |  |  |
| Recruiting a job candidate | E |  |  |  |
| Monitoring the quality standards for food safety and hygiene | C |  |  |  |
| Repairing malfunctioning electrical equipment | R |  |  |  |
| Carrying out laboratorial analyses | I |  |  |  |
| Designing a poster for an exhibition | A |  |  |  |
| Helping others with their personal problems | S |  |  |  |
| Organising a conference | E |  |  |  |
| Preparing financial reports | C |  |  |  |
| Being responsible for the maintenance of IT hardware | R |  |  |  |
| Analysing statistics | I |  |  |  |
| Designing webpages | A |  |  |  |
| Developing council prevention campaigns | S |  |  |  |
| Presenting new policy propositions | E |  |  |  |
| Collecting quantitative and qualitative data | I |  |  |  |
| Develop new methods for industrial production | R |  |  |  |
| Treating diseases in animals | I |  |  |  |
| Editing the sound and images for a movie | A |  |  |  |
| Formulating education and training policies | S |  |  |  |
| Drawing up the budgets | C |  |  |  |
| Doing the follow up on building sites | R |  |  |  |
| Analysing x-rays/brain scans | I |  |  |  |
| Fit out a show room | A |  |  |  |
| Sport guidance for children, the elderly, … | S |  |  |  |
| Formulate a theory about the differences between population groups | I |  |  |  |
| Monitor quality standards | C |  |  |  |
| Maintaining airplanes | R |  |  |  |
| Investigating the impact of historical people | A |  |  |  |
| Composing a work of music | A |  |  |  |
| Providing guidance for victims | S |  |  |  |
| Selling a product or service | E |  |  |  |
| Calculating prices | C |  |  |  |
| Installing and maintaining computer servers | R |  |  |  |
| Designing an advertising folder | A |  |  |  |
| Providing information about the assistance for the poor | S |  |  |  |
| Drawing up an organisational business or policy plan | E |  |  |  |
| Checking bank transactions | C |  |  |  |
| Developing windmill parks | R |  |  |  |
| Prove a theorem | I |  |  |  |
| Analysing text structures | A |  |  |  |
| Giving travel advice | S |  |  |  |
| Negotiating contracts | E |  |  |  |
| Drawing up a contract | C |  |  |  |
| Investigating chromosomal defects | I |  |  |  |
| Writing scenarios | A |  |  |  |
| Holding tests, questionnaires and in-depth interviews | S |  |  |  |
| Screening the administration | C |  |  |  |
| Working on a drilling rig | R |  |  |  |
| Turning an idea into a film | A |  |  |  |
| Giving care to patients | S |  |  |  |
| Restructuring an organisation or company | E |  |  |  |
| Checking the compliance of regulations | C |  |  |  |
| Excluding alternative explanations through experiments | I |  |  |  |
| Designing the layout of a hospital | A |  |  |  |
| Advising youngsters regarding their vocational choice | S |  |  |  |
| Exploring new economic markets | E |  |  |  |
| Drawing up the annual report | C |  |  |  |
| Setting up a festival stage | R |  |  |  |
| Developing a new medicine | I |  |  |  |
| Writing a review | A |  |  |  |
| Giving training in communication skills | S |  |  |  |
| Starting up an enterprise | E |  |  |  |
| Investigating a cost structure | C |  |  |  |
| Creating a technical drawing | R |  |  |  |
| Putting theories in their historical and social context | I |  |  |  |
| Creating an art piece | A |  |  |  |
| Giving health advice | S |  |  |  |
| Giving health and parenting education | E |  |  |  |
| Calculating expenses | C |  |  |  |
| Disassembling electrical appliances | R |  |  |  |
| Comparing cultures | A |  |  |  |
| Guiding minority groups on the job market | S |  |  |  |
| Conducting a meeting | E |  |  |  |
| Drawing up a timetable | C |  |  |  |
| Measuring a lane | R |  |  |  |
| Supporting and following up foster families | S |  |  |  |
| Attracting sponsors | E |  |  |  |
| Standing in front of a classroom | S |  |  |  |
| Leading a team | E |  |  |  |
| Managing a database | C |  |  |  |
| Collecting soil samples | R |  |  |  |
| Beginning a herbarium (a plant collection) | I |  |  |  |
| Counseling underprivileged people | S |  |  |  |
| Formulating a treatment plan | S |  |  |  |
| Studying the physical endurance of athletes | I |  |  |  |
| **B** |  |  |  |  |
| Occupations | Dimension | YES | NO |  |
| Industrial designer | R |  |  |  |
| Civil engineer | I |  |  |  |
| Fashion designer | A |  |  |  |
| Policy advisor in political and international relations | E |  |  |  |
| Recruitment and selection advisor | E |  |  |  |
| Damage expert | C |  |  |  |
| Agricultural technician | R |  |  |  |
| Teacher | S |  |  |  |
| Business economist | C |  |  |  |
| Accountant | C |  |  |  |
| Electrical engineer | R |  |  |  |
| Biologist | I |  |  |  |
| Art/music teacher | A |  |  |  |
| Speech therapist | S |  |  |  |
| Bank manager | C |  |  |  |
| Landscape architect | R |  |  |  |
| Physicist | I |  |  |  |
| Editor | A |  |  |  |
| Student counselor | S |  |  |  |
| Tax supervisor | C |  |  |  |
| Neurologist | I |  |  |  |
| Policy advisor art and culture | A |  |  |  |
| Educator | S |  |  |  |
| Marketing manager | E |  |  |  |
| Safety advisor | C |  |  |  |
| Construction manager | R |  |  |  |
| Historian | I |  |  |  |
| Director | A |  |  |  |
| Communication manager | E |  |  |  |
| Manager (of a company) | E |  |  |  |
| Judge | C |  |  |  |
| Forester | R |  |  |  |
| Researcher | I |  |  |  |
| Graphic designer | A |  |  |  |
| Psychologist | S |  |  |  |
| Lawyer | E |  |  |  |
| Notary | C |  |  |  |
| Mathematician | I |  |  |  |
| Art historian | A |  |  |  |
| Social worker | S |  |  |  |
| Politician | E |  |  |  |
| Pilot | R |  |  |  |
| Pharmacist | I |  |  |  |
| Linguist | A |  |  |  |
| Divorce mediator | S |  |  |  |
| Journalist | A |  |  |  |
| Structural engineer | R |  |  |  |
| Lab assistant | I |  |  |  |
| Photographer | A |  |  |  |
| Nurse | S |  |  |  |
| Advertising campaign manager | E |  |  |  |
| Chemist | I |  |  |  |
| Tax specialist | C |  |  |  |
| Architect | R |  |  |  |
| Artist | A |  |  |  |
| Educational scientist | S |  |  |  |
| Librarian | A |  |  |  |
| Philosopher | I |  |  |  |
| Representative | E |  |  |  |
| Geneticist | I |  |  |  |
| Interior designer | A |  |  |  |
| Estate agent | E |  |  |  |
| Physiotherapist | S |  |  |  |
| Meteorologist | I |  |  |  |
| Sales manager | E |  |  |  |
| Statistician | I |  |  |  |

Note. Dimensions were masked for the participant and the instructions were as follows. A: Mark the YES column for activities you enjoy to do or activities you would like to try. Mark the NO column for activities you would not like to do. If you really don’t know what the activity implies, skip the item. B: Mark YES for professions you would like to practice or that you would like to try. Mark NO for professions you would not like to do. If you think a little bit, you probably know most professions. If you really don’t know what a profession entails, skip the item.
